# Corrigenda: De Mattia W, Neiber MT, Groh K (2018) Revision of the genus-group *Hystricella* R. T. Lowe, 1855 from Porto Santo (Madeira Archipelago), with descriptions of new recent and fossil taxa (Gastropoda, Helicoidea, Geomitridae). ZooKeys 732: 1–125. https://doi.org/10.3897/zookeys.732.21677

**DOI:** 10.3897/zookeys.733.23906

**Published:** 2018-02-01

**Authors:** Willy De Mattia, Marco T. Neiber, Klaus Groh

**Affiliations:** 1 Natural History Museum Vienna, Burgring 7, 1010 Vienna, Austria; 2 International Centre for Genetic Engineering and Biotechnology, Padriciano 99, 34149, Trieste, Italy; 3 Centre of Natural History, Zoological Museum, University of Hamburg, Martin-Luther-King-Platz 3, D-20146 Hamburg, Germany; 4 Hinterbergstraße 15, D-67098 Bad Dürkheim, Germany

We proposed the name *Wollastonia* De Mattia, Neiber & Groh, 2018 for a small group of land snails that are endemic to the island of Porto Santo and some of its sourrounding islet in the Madeiran Archipelago (De Mattia et al. 2018). Unfortunately, and only explicable as the consequence of a whole chain of small mistakes or omissions, it slipped our attention that the name is a junior homonym of *Wollastonia* Heer, 1852 (Coleoptera), *Wollastonia* Horn, 1873 (Coleoptera) and *Wollastonia* Machado, 1984 (Coleoptera) (see Heer 1852: 13, Horn 1873: 433–434, Machado 1984: 131). Consequently, the genus needs a new name and we herewith propose *Wollastonaria* nom. n. as a new replacement name for *Wollastonia* De Mattia, Neiber & Groh, 2018. The type species of *Wollastonaria* nom. n. is the nominal species *Helix* [*Helicella*] *turricula* R. T. Lowe, 1831. A detailed description of the genus is given in De Mattia et al. (2018) under the name *Wollastonia*. Included are the following species and subspecies: *Wollastonaria turricula* (R. T. Lowe, 1831), comb. n., *Wollastonaria vermetiformis* (R. T. Lowe, 1855), comb. n., *Wollastonaria ripkeni* (De Mattia & Groh, 2018), comb. n., *Wollastonaria falknerorum* (Groh, Neiber & De Mattia, 2018), comb. n., *Wollastonaria leacockiana* (Wollaston, 1878), comb. n., *Wollastonaria beckmanni* (De Mattia & Groh, 2018), comb. n., *Wollastonaria jessicae jessicae* (De Mattia, Neiber & Groh, 2018), comb. n., *Wollastonaria jessicae monticola* (De Mattia, Neiber & Groh, 2018), comb. n., *Wollastonaria klausgrohi* (De Mattia & Neiber, 2018), comb. n., *Wollastonaria oxytropis* (R. T. Lowe, 1831), comb. n., *Wollastonaria subcarinulata* (Wollaston, 1878), comb. n., and *Wollastonaria inexpectata* (De Mattia & Groh, 2018), comb. n.

Fig. 15 should read Caseolus (Leptostictea) leptosticus, Ponta do Garajau, Madeira.

Fig. 16 should read Caseolus (Helicomela) punctulatus punctulatus, Fonte da Areia.
